# Nano-energy interference: A novel strategy for blunting tumor adaptation and metastasis

**DOI:** 10.1016/j.mtbio.2024.100984

**Published:** 2024-02-03

**Authors:** Fei Teng, Dong Fu, Chen-Cheng Shi, An Xiong, Meng-Xuan Yang, Chang Su, Ming Lei, Yi-Ou Cao, Xiao-Dong Shen, Yi Chen, Pu-Hua Wang, Shao-Qun Liu

**Affiliations:** a Department of Gastrointestinal Surgery, Minhang Hospital, Fudan University, Shanghai, 201199, PR China; b Key Laboratory of Whole-period Monitoring and Precise Intervention of Digestive Cancer (SMHC), Minhang Hospital & AHS, Fudan University, Shanghai, 201199, PR China; c Department of Pediatric Orthopedics, Children's Hospital of Fudan University, National Children's Medical Center, Shanghai, 201102, PR China

**Keywords:** Tumor adaptation, Energy metabolism, Mitochondrial respiration, Ribosome inhibition, Lipid nanoparticles

## Abstract

Blunting the tumor's stress-sensing ability is an effective strategy for controlling tumor adaptive survival and metastasis. Here, we have designed a cyclically amplified nano-energy interference device based on lipid nanoparticles (LNP), focused on altering cellular energy metabolism. This innovative nano device efficiently targets and monitors the tumor's status while simultaneously inhibiting mitochondrial respiration, biogenesis and ribosome production. To this end, we first identified azelaic acid (AA), a binary acid capable of disrupting the mitochondrial respiratory chain. Upon encapsulation in LNP and linkage to mitochondrial-targeting molecules, this disruptive effect is further augmented. Consequently, tumors exhibit a substantial upregulation of the glycolytic pathway, intensifying their glucose demand and worsening the tumor's energy-deprived microenvironment. Then, the glucose analog, 2-Deoxy-D-glucose (2-DG), linked to the LNP, efficiently targets tumors and competitively inhibits the tumor's normal glucose uptake. The synergetic results of combining AA with 2-DG induce comprehensive energy deficiency within tumors, blocking the generation of energy-sensitive ribosomes. Ultimately, the disruption of both mitochondria and ribosomes depletes energy supply and new protein-generating capacity, weakening tumor's ability to adapt to environmental stress and thereby inhibiting growth and metastasis. Comprehensively, this nano-energy interference device, by controlling the tumor's stress-sensing ability, provides a novel therapeutic strategy for refractory tumors.

## Graphical abstract

Our research presents a lipid nanoparticle-based nano-energy interference device designed to disrupt tumor energy metabolism. The device encapsulates azelaic acid, augmenting mitochondrial disruption and intensifying tumor energy deficiency. Additionally, the device binds with the glucose analog, 2-deoxy-D-glucose, inhibiting tumor glucose uptake. The sustained release of azelaic acid induces comprehensive energy deficiency in tumors, impeding ribosome generation. This innovative strategy offers a promising approach to the treatment of refractory tumors.

**Figure:**
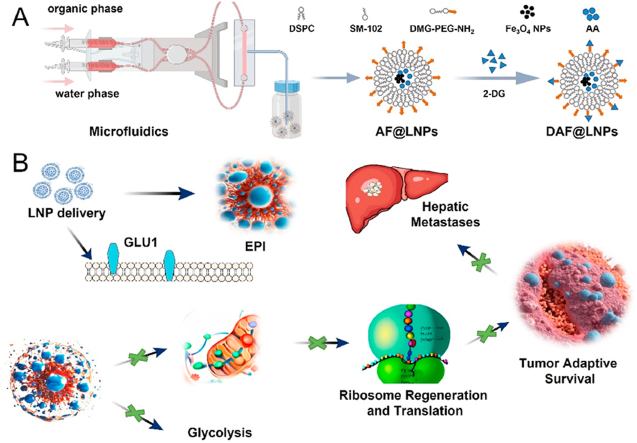

## 1 Introduction

Colorectal cancer (CRC), the third most common cancer globally, poses a formidable challenge to healthcare systems due to its elevated mortality rate, primarily attributed to metastasis [1,2]. The liver is the most frequent site for metastasis, representing more than 70 % of cases, and it significantly worsens the prognosis. This metastasis decreases the 5-year survival rate from over 90 % to less than 15 % in the cases of metastatic CRC [[3], [4], [5], [6]]. Thus, the prevention and treatment of liver metastasis play a pivotal role in improving patient survival and overall quality of life. However, CRC is a complex heterogeneous disease influenced by the diverse of genetic, epigenetic and environmental factors that collectively shape its development and progression [[7], [8], [9]]. Over the past few decades, significant progress has been achieved in understanding the molecular mechanisms underlying CRC, thereby offering numerous novel intervention targets for clinical treatment. Mutations in pivotal genes such as APC, KRAS and TP53 genes have been identified as key driving factors in CRC [[10], [11], [12]]. Moreover, epigenetic changes, including DNA methylation and histone modifications, have been implicated in the pathogenesis of CRC [[13], [14], [15]]. These findings have not only deepened our comprehension of CRC but have also paved the way for the development of targeted therapies. However, addressing metastatic CRC remains challenging at present. On one hand, the tumor exhibits remarkable adaptability, characterized by resistance to apoptosis and the ability to survive under adverse conditions [16]. On the other hand, the effectiveness of targeted therapy is constrained by genetic heterogeneity and drug resistance, placing limitations on conventional treatments in clinical practice [17]. Therefore, the development of new therapeutic modalities is particularly urgent. Recent research has focused on the reshaping of energy metabolism in tumor cells, revealing alterations in both energy production and nutrient utilization as critical factors in the progression and metastasis of CRC [18,19]. The intricacy and flexibility of cancer reside in the tumor's capacity to perceive and respond to stress, maintain energy equilibrium, and efficiently synthesize proteins, all of which are vital for its adaptability and survival in shifting circumstances. Nevertheless, exploiting these characteristics for therapeutic endeavors remains a formidable challenge at present, as methods to overcome the intricacy of tumors and their remarkable adaptability have yet to be reported.

The adaptive survival and metastasis of tumors are not merely a result of random genetic mutations. Instead, they are orchestrated by a complex network of cellular processes centered around energy metabolism [20,21]. The tumor's ability to maintain energy homeostasis, even under stress conditions, critically influences its survival and progression. Mitochondria, commonly referred to as the powerhouses of the cell, play a pivotal role in this context, responsible not only for adenosine triphosphate (ATP) production but also in regulating cellular processes such as apoptosis and signal transduction [22,23]. Mitochondria serve as primary sites for aerobic respiration, converting glucose and oxygen into ATP, the cell's energy currency [24]. However, cancer cells, in contrast to normal cells, exhibit a distinctive metabolic phenotype known as the Warburg effect. This phenomenon involves a shift from oxidative phosphorylation to glycolysis, even under oxygen-rich conditions, facilitating rapid proliferation and survival in hypoxic tumor microenvironments [25,26]. Beyond glucose, recent studies highlight the adaptability of cancer cells in utilizing diverse substrates like glutamine, fatty acids, and lactate to fuel their energy metabolism [[27], [28], [29]]. This metabolic flexibility allows cancer cells to adapt to nutrient-poor conditions and contributes to their survival, growth, and metastasis [30,31].

Concurrently, ribosomes, the cellular protein factories, are integral to protein synthesis crucial for tumor growth and survival [32,33]. As an energy-intensive process, the ribosomal function is susceptible to the cell's energy status [[34], [35], [36]]. Their function and efficiency can be significantly affected under conditions of energy deficiency. This sensitivity to energy status underscores the importance of maintaining energy homeostasis for the survival and progression of tumors.

Based on the research mentioned above, the interplay among these components-mitochondria, ribosomes, and the tumor's versatile energy metabolism-provides the foundation for the tumor's adaptive reshaping. Consequently, this interplay presents a unique therapeutic target that could potentially open new directions in cancer treatment. However, to date, research reports are scarce in this particular field. On one hand, targeting the energy metabolism of tumors is not straightforward. It requires a thorough understanding of the tumor's metabolic flexibility and the complex network of cellular processes that support its survival and progression. This understanding is crucial for the development of effective therapeutic strategies that can disrupt the tumor's energy metabolism without causing significant harm to normal cells. On the other hand, the challenge lies not only in identifying the key players in the tumor's energy metabolism but also in devising innovative strategies to target them. The rapid advancement of nanotechnology, particularly the successful clinical application of lipid nanoparticles (LNPs), provides an ideal nano vector for the synergistic delivery of drugs [[37], [38], [39], [40]]. Fe_3_O_4_ is a common magnetic material known for its capability to influence magnetic resonance imaging (MRI) signal intensity [41,42]. By incorporating Fe_3_O_4_, LNPs have gained the ability for MRI imaging to monitor tumors effectively. MRI enables non-invasive detection of the in vivo distribution of LNPs, allowing real-time quantitative monitoring of changes in tumor growth. This provides a crucial means of tracking for LNPs as a novel approach to cancer treatment. Therefore, we have delved into the uncharted territory of the tumor's energy metabolism and presented a groundbreaking therapeutic strategy based on nanotechnology that has the potential to revolutionize cancer treatment.

In our study, we delve into this uncharted territory and offer a pioneering approach that exploits the tumor's reliance on mitochondrial energy production and ribosomal protein synthesis. We discovered that Azelaic acid (AA), a naturally occurring dicarboxylic acid [43], disrupts the mitochondrial respiratory chain, thereby impairing energy production. Building on this discovery, we developed a novel nano-energy interference device. We encapsulated AA in liposomes and linked it with a glucose analog, 2-Deoxy-D-glucose (2-DG) [44]. This design not only targets the tumor's energy production but also inhibits its ability to uptake glucose, thereby inducing a state of energy deficiency. This dual-action approach disrupts the tumor's adaptive survival and metastasis, offering a promising therapeutic strategy for refractory tumors. After Fe_3_O_4_ is incorporated, the final LNPs not only intervene in the energy metabolism of tumors to inhibit their growth but also enable MRI monitoring through Fe_3_O_4_. Our findings underscore the potential of targeting the tumor's energy metabolism as a promising approach for cancer therapeutics.

## 2 Results and discussion

### 2.1 Characteristics of nano-energy interference

**Fig. 1:**
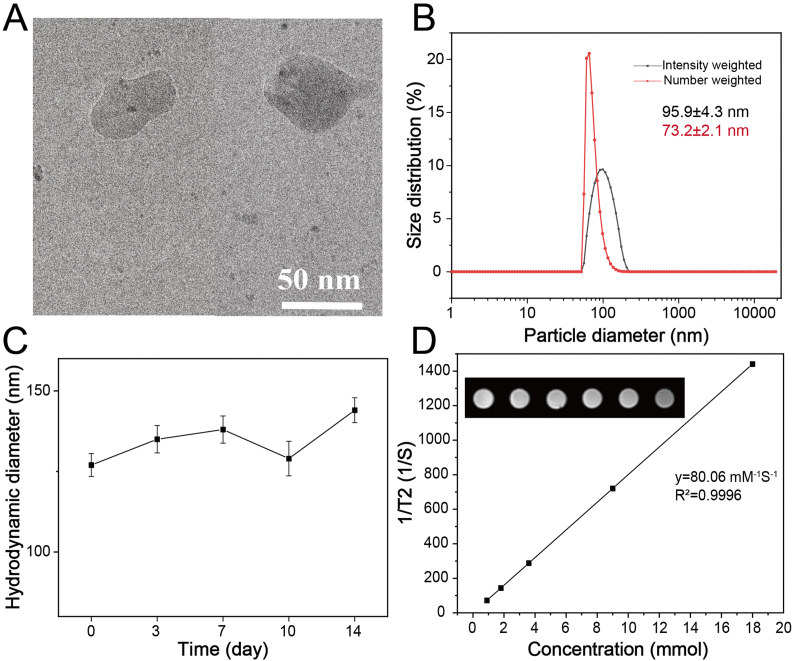
Preparation and characterization of nanosystem DAF@LNPs. (A) Transmission electron microscopy (TEM) reveals a homogeneous morphology of DAF@LNPs, exhibiting an average diameter of approximately 55 nm. (B) Dynamic light scattering (DLS) analysis validates the size distribution of DAF@LNPs, including intensity weighted (%) and number weighted (%) respectively. (C) Stability assessment of DAF@LNPs conducted in cell culture medium, supplemented with 10 % fetal bovine serum, demonstrates sustained stability over a 14-day period. (D) T2-weighted magnetic resonance (MR) imaging showcases the concentration-dependent darkening impact of DAF@LNPs, with a calculated transverse relaxation rate (r2) of 80.06 mM^−1^ s^−1^.

The nano-energy interference (DAF@LNPs) was constructed based on the lipid nanoparticle that co-loaded Fe_3_O_4_ NPs/AA and was modified with 2-DG, synthesized using microfluidic technology as previously described [45]. Transmission electron microscopy (TEM) analysis revealed that DAF@LNPs exhibit a uniform morphology, with an average diameter of approximately 50 nm (Fig. 1A and Supplementary Fig. S1). This finding was further confirmed by dynamic light scattering (DLS) examination (Fig. 1B). Using atomic absorption spectroscopy and high-performance liquid chromatography (HPLC), the loading efficiency of iron or AA was determined to be approximately 5.37 wt% and 1.64 wt%, respectively, indicating successful loading of Fe_3_O_4_ NPs and AA into LNPs. Through mass spectrometry analysis, a comparison was made between free 2-DG, LNPs without loaded 2-DG (AF@LNPs), and LNPs loaded with 2-DG (DAF@LNPs). The results indicate that 2-DG has been successfully modified on the LNP (Supplementary Fig. S2). Also, the stability of DAF@LNPs was evaluated using DLS, which showed no significant changes in hydrodynamic size over 14 days in the cell culture medium containing 10 % fetal bovine serum (FBS) (Fig. 1C). These results provide preliminary evidence for the suitability of DAF@LNPs for further biological research. Additionally, LNPs unmodified with 2-DG (abbreviated as AF@LNPs) and fluorescently labeled LNPs (termed as Fluro-DAF@LNPs) were also prepared under the same condition for subsequent biological investigation. Given the paramagnetic nature of Fe_3_O_4_ nanoparticles, we subsequently investigated the magnetic resonance imaging capabilities of DAF@LNPs. The T2-weighted MR images of DAF@LNPs exhibited a concentration-dependent darkening effect, with a calculated transverse relaxation rate (r2) of 80.06 mM^−1^ s^−1^ (Fig. 1D).

### 2.2 Cellular uptake and mitochondrial targeting of nano-energy interference

**Fig. 2:**
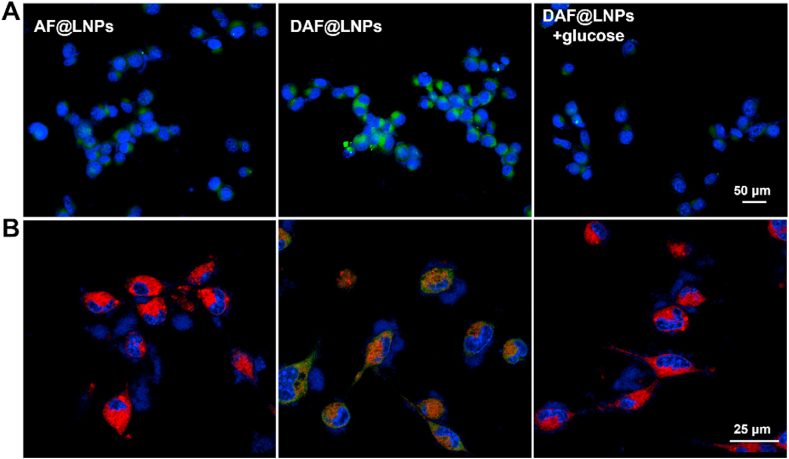
Phagocytosis of nanosystems (DAF@LNPs) by cells and characterization of targeting the endoplasmic reticulum in vitro. (A) Confocal microscopy images depicting HCT116 cells incubated with Fluro-DAF@LNPs and Fluro-AF@LNPs. (B) Confocal imaging reveals the co-localization of Fluro-DAF@LNPs (green) with mitochondria (red) in HCT116 cells, indicating the precise targeting of mitochondria through 2-DG-coupled nano-energy interference. (For interpretation of the references to color in this figure legend, the reader is referred to the Web version of this article.)

Tumor cells exhibit a phenomenon known as the Warburg effect, a metabolic phenomenon marked by aerobic glycolysis and heightened glucose consumption. Concurrently, there is an upregulation in the expression of glucose transporter-1 (GLUT-1) on the cell surface. As a glucose analog, 2-DG enters cells via GLUT-1, similar to glucose. It acts as a competitive inhibitor of glycolysis by binding with hexokinase, a key enzyme in the glycolytic pathway. Given the structural resemblance between 2-DG and glucose, and considering the strong dependency of tumor cells on glucose, it is plausible that our 2-DG-coupled nanocompiler would be selectively taken up by tumors and actively participate in energy metabolism. To this end, we evaluated the targeting effect of 2-DG-coupled nano-energy interference using the colon cancer cell line HCT116, which has been previously confirmed to have high levels of GLUT-1 expression and glucose consumption. After incubation with FITC-labeled LNPs (50 μg Fe/mL) for 3 h, the cellular uptake of 2-DG-coupled nano-energy interference in HCT116 cells was detected through confocal imaging. Compared to the non-targeted Fluro-AF@LNPs group, a strong fluorescence signal was observed in cells treated with Fluro-DAF@LNPs (Fig. 2A). However, with the addition of extra glucose, the fluorescence signal significantly decreased (Fig. 2A), suggesting that 2-DG-coupled nano-energy interference exhibited specific tumor-targeting characteristics. Consistent with the results of fluorescence observation, flow cytometry further confirmed the specific uptake and internalization of Fluro-DAF@LNPs in HCT116 cells (Supplementary Fig. S3).

Previous research has indicated that AA can participate in certain energy metabolism pathways [46], with its intermediates also taking part in the tricarboxylic acid cycle. Given its cell-targeting effects, we further sought to validate the efficacy of our nano-energy interference targeting method in penetrating intracellular barriers, specifically targeting mitochondria, and achieving precise organelle tracking. To investigate the intracellular localization of DAF@LNPs post-internalization, cells were subjected to a 12-h incubation with FITC-labeled LNPs (50 μg Fe/mL), followed by co-staining with MitoTracker Red to determine the spatial relationship between LNPs and mitochondria. The cell nuclei were concomitantly stained with 4′,6-diamidino-2-phenylindole (DAPI). Confocal laser imaging revealed a distinct co-localization (displayed in yellow) between Fluro-DAF@LNPs (green) and mitochondria (red) (Fig. 2B), suggesting the primary targeting of Fluro-DAF@LNPs to mitochondria. In contrast, the co-localization of non-targeted Fluro-AF@LNPs with mitochondria was significantly diminished (Fig. 2B). These findings indicate that 2-DG-coupled LNPs could be internalized by HCT116 cells, effectively delivering AA to mitochondria, thereby laying the groundwork for subsequent regulation of tumor metabolic remodeling.

### 2.3 Nano-energy interference reshapes tumor metabolism and organelle quality control

In light of the encouraging results obtained, we proceeded to assess the physiological functions of LNPs. Azelaic acid, an aliphatic dicarboxylic acid, is typically present in trace amounts in the human body. While not deemed an essential physiological substance, AA plays a role in certain physiological and metabolic processes. Previous investigations have shown that AA could participate in energy metabolism pathways, wherein it undergoes metabolization into acetone or other intermediates [47]. These intermediates further participate in the tricarboxylic acid cycle, thereby contributing to energy generation. Despite its energy generation being comparatively less efficient than that of metabolites like glucose, AA can provide cells with additional energy under specific conditions. Additionally, AA could serve as an intermediate in drug preparation, with recent studies potentially involving its potential in synthesizing new derivatives for treatment-specific diseases or enhancing drug stability and bioavailability. These findings underscore AA's excellent biocompatibility and its role in certain physiological regulatory functions. Nevertheless, the impact of AA on tumors remains largely unexplored.

**Fig. 3:**
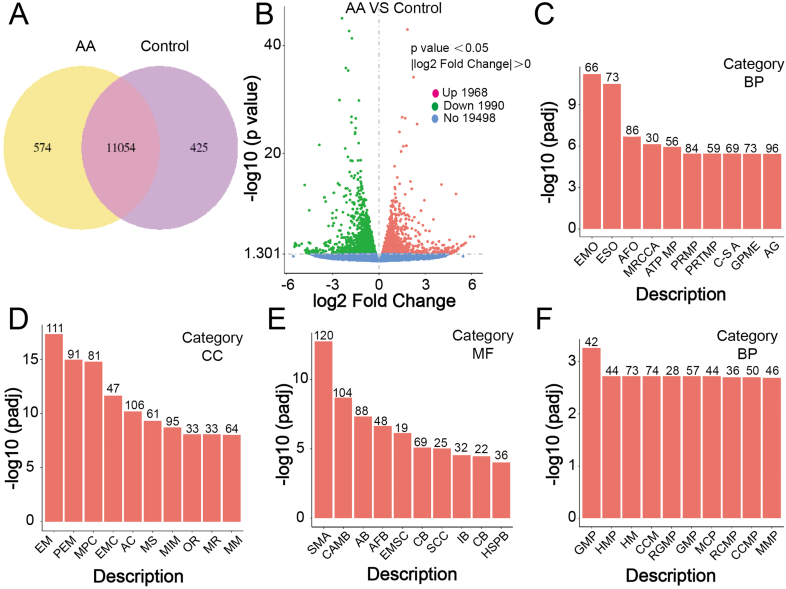
Transcriptomics analysis of tumor cells treated with DAF@LNPs. (A) The Venn diagram visually presents the distinct and shared gene expression patterns in tumor cells treated with DAF@LNPs, delineating the number of genes uniquely expressed in each group/sample. Overlapping regions signify co-expressed genes in two or more groups/samples. (B) The volcano plot illustrates the differential expression of genes in tumor cells post DAF@LNPs treatment. Red points depict upregulated genes, while downregulated genes are represented by green points, emphasizing the notable differences in gene expression between the treatment and control groups. (C) Gene ontology (GO) enrichment analysis of biological processes demonstrates the downregulation of functions associated with the mitochondrial respiratory chain in tumor cells subsequent to DAF@LNPs treatment. (D) GO enrichment analysis of cellular components reveals a significant decrease in mitochondrial components in tumor cells treated with DAF@LNPs. (E) GO enrichment analysis of molecular functions highlights the downregulation of ATP generation in tumor cells following DAF@LNPs treatment. (F) The inhibition of ribosome biogenesis in tumor cells after DAF@LNPs treatment suggests a substantial impact on energy-consuming physiological processes. In Fig. 3C–F, the gene enrichment abbreviations on the x-axis correspond to those displayed in Table S1. (For interpretation of the references to color in this figure legend, the reader is referred to the Web version of this article.)

Against this backdrop, we utilized transcriptomics as the primary method to conduct an exhaustive analysis of tumor cells subjected to various treatments. This approach aimed to explore the tumor regulatory capacity of LNPs loaded with AA. The Venn diagram illustrating co-expression showed the number of genes uniquely expressed in each group/sample, with overlapping regions demonstrating the genes co-expressed in two or more groups/samples (Fig. 3A). Complementing this, the volcano plot provided an intuitive display of the distribution of differential genes in each comparison combination (Fig. 3B). Red points signify upregulated genes, whereas green points denote downregulated genes. Comprehensively, these results revealed that post DAF@LNPs treatment, 1968 genes were upregulated, and 1990 genes were downregulated in tumor cells compared to the control group. This observation indicated significant differences in gene expression between groups. Subsequently, transcriptomics gene ontology (GO) enrichment was performed to evaluate the biological processes, cellular components, and molecular functions (Fig. 3C–E). The results revealed that after DAF@LNPs treatment, relevant functions related to the mitochondrial respiratory chain, mitochondrial components, and ATP generation in tumor cells were significantly downregulated. This suggested that mitochondrial respiration and component generation were important targets of DAF@LNPs. Interestingly, the expression of the tumor's glycolysis-related gene set was increased considerably. We hypothesize that this surge is a compensatory response to robustly inhibited tumor aerobic respiration, prompting the tumor to rely on glycolysis for energy acquisition. This is a less efficient energy conversion method that requires tumor cells to intake more fuel. The scarcity of the tumor microenvironment exacerbated the tumor's energy crisis, posing a formidable challenge to its survival. Furthermore, our observations indicated a substantial inhibition of ribosome biogenesis in tumor cells at this time (Fig. 3F). Given that ribosome generation and translation represent the most energy-consuming physiological processes in organisms, their sensitivity to the energy deficiency of tumors was pronounced.

### 2.4 Nano-energy interference disrupts mitochondrial function and enhances glucose uptake

**Fig. 4:**
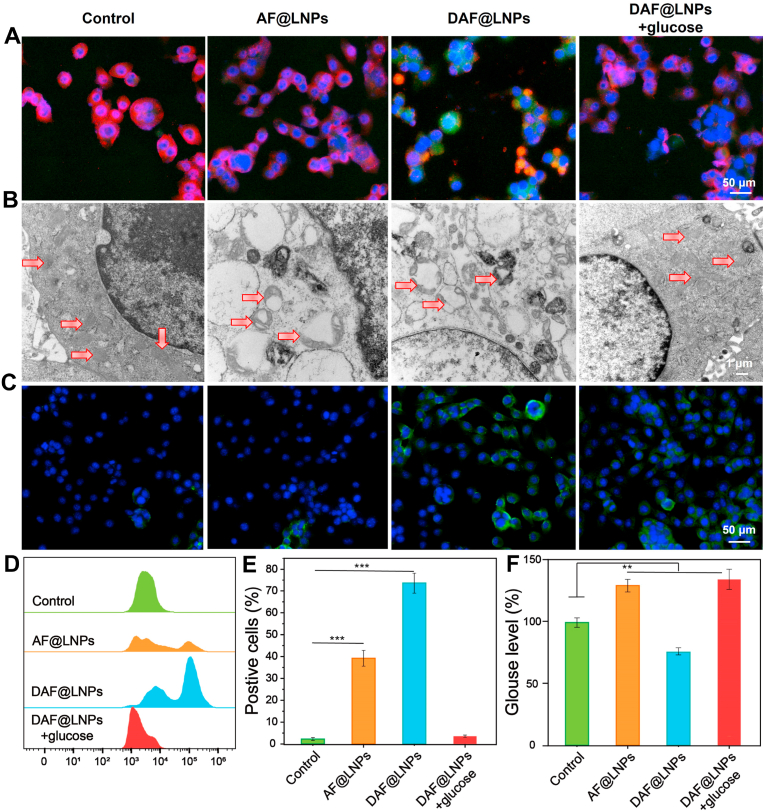
Impact of DAF@LNPs on tumor mitochondria and oxidative stress. (A) JC-1 staining reveals the disruption of mitochondrial membrane potential in tumor cells following various treatments. Notably, DAF@LNPs treatment induces a more pronounced green fluorescence, indicating a further deterioration of mitochondrial function. (B) Cellular electron microscopy images depict the structural disruption of mitochondria in tumor cells post-treatment. DAF@LNPs treatment intensifies mitochondrial structural disorder, pointing to a targeted effect on mitochondria. (C) Evaluation of intracellular reactive oxygen species (ROS) levels using 2′,7′-dichlorodihydrofluorescein diacetate (DCFH-DA) staining. DAF@LNPs treatment results in more intense fluorescence, signifying increased ROS production compared to other treatments. (D) Flow cytometry analysis confirms the heightened intracellular ROS levels in tumor cells treated with DAF@LNPs, as evidenced by increased DCF fluorescence. (E) A quantitative assessment of ROS fluctuations following diverse treatments. (F) Intracellular glucose levels in tumor cells under different treatment conditions. DAF@LNPs competitively inhibit glucose uptake, leading to a reduction in intracellular glucose levels and exacerbating the energy-deficient state of the tumor. (For interpretation of the references to color in this figure legend, the reader is referred to the Web version of this article.)

To validate the accuracy of the above-mentioned omics results, we proceeded to assess the disruptive impact of DAF@LNPs on tumor mitochondria and the resultant oxidative stress. Following various treatments, the mitochondrial status was first evaluated via JC-1 staining. Compared to the control group, both AF@LNPs and DAF@LNPs + glucose treatments led to a significant increase in green fluorescence intensity (Fig. 4A), indicative of mitochondrial membrane potential disruption. Interestingly, DAF@LNPs treatment, which presented a targeted effect, induced a more pronounced green fluorescence than others, suggesting further deterioration of mitochondrial function. Then, cellular electron microscopy images further illustrated the extent of mitochondrial structural disruption (Fig. 4B). Compared to the control group, both treatments involving DAF@LNPs + glucose and AF@LNPs led to significant mitochondrial structural disorder. This disrupting effect was further intensified with DAF@LNPs, owing to its distinctive mitochondrial targeting capability (Fig. 4B).

Next, we tested the tumor's ability to cope with oxidative stress at this time. For this purpose, cells were treated and subsequently stained with 2′,7′-dichlorodihydrofluorescein diacetate (DCFH-DA), a compound that engages with reactive oxygen species (ROS) to yield 2′,7′-dichlorodihydrofluorescein (DCF). It is well known that the fluorescence intensity of DCF correlates with an elevation in intracellular ROS levels. Our observations showed that compared to the control group, cells treated with AF@LNPs displayed a significantly enhanced fluorescence signal. This effect was further amplified in cells subjected to mitochondrial-targeting DAF@LNPs, exhibiting a more pronounced fluorescence intensity (Fig. 4C). These findings were also confirmed through flow cytometry analysis (Fig. 4D and E).

Inspired by the impressive mitochondrial interference capabilities demonstrated by nano-energy interference, our subsequent research involved analyzing alterations in intracellular glucose concentration in tumor cells under different treatment conditions. This objective was to confirm whether such interventions would activate tumor glycolysis compensation, as shown in the omics results. Following a 12-h incubation in media containing AF@LNPs, DAF@LNPs, or DAF@LNPs + glucose, respectively, intracellular glucose levels were assessed using a glucose assay kit. The results showed that both AA and AF@LNPs could lead to an increase in glucose uptake. Notably, the addition of the competitive molecule 2-DG resulted in a reduction in intracellular glucose levels mediated by DAF@LNPs (Fig. 4F). This implies that the disruption of mitochondrial function alone could increase the glucose demand of tumors, while DAF@LNPs competitively inhibit glucose uptake in cancer cells, effectively reducing intracellular glucose levels within the tumor. Such a mechanism would further exacerbate the energy-deficient state of the cancer.

**Fig. 5:**
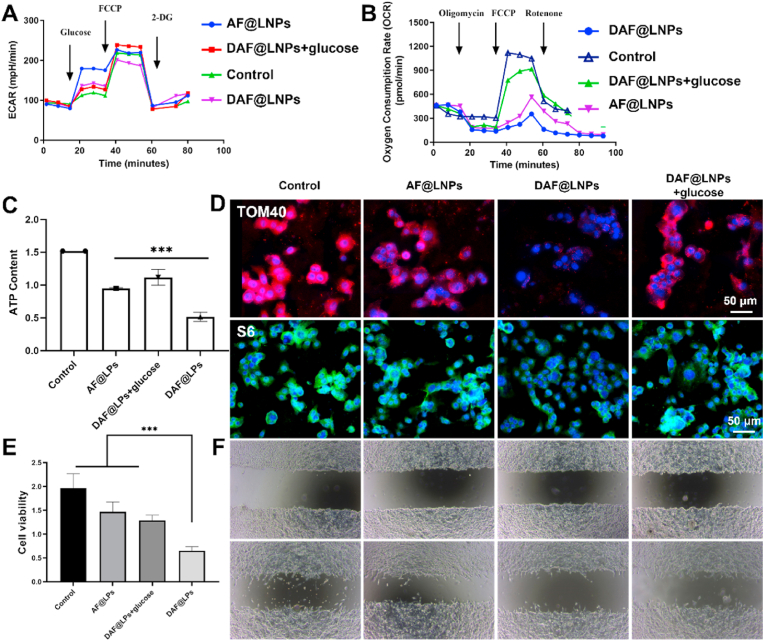
Impact of DAF@LNPs on tumor bioenergetics, mitochondrial and ribosomal quality control, as well as tumor cell viability. (A) Profiling of tumor cell bioenergetics using the Seahorse XFe96 analyzer following treatment with AA, AF@LNPs, or DAF@LNPs, respectively. AA and AF@LNPs treatments result in a partial increase in glycolytic capacity and acidification rate, whereas DAF@LNPs treatment induces a rapid decline in glycolytic capacity. (B) Assessment of basal oxygen consumption rate (OCR), maximal respiration, and ATP production in tumor cells post-treatment. AA and AF@LNPs treatments reduce cellular basal OCR, maximal respiration, and ATP production, with these effects intensifying upon DAF@LNPs treatment. (C) Quantitative analysis of OCR and maximal respiration in tumor cells under various treatment conditions, highlighting a significant increase in basal OCR and maximal respiration with DAF@LNPs treatment. (D) Immunoblot analysis of TOM40 and S6 expression levels in tumor cells post-treatment. DAF@LNPs effectively inhibit the production of mitochondria and ribosomes in the tumor, whereas other treatments only partially inhibit these processes. (E) Cell viability assessment using the CCK-8 assay after a 48-h incubation period. DAF@LNPs exhibit significant cytotoxicity, while AA and AF@LNPs only show partial inhibition of tumor cells. (F) Cell scratch assay demonstrating the impact of various treatments on tumor cell migration. DAF@LNPs effectively inhibit tumor cell migration, whereas AA and AF@LNPs only partially inhibit tumor migration speed.

To underscore the effectiveness of nanoparticle-induced mitochondrial disruption in inducing cellular energy deficiency, we employed the Seahorse XFe96 Analyzer to profile the bioenergetics of tumor cells. The application of both DAF@LNPs + glucose and AF@LNPs resulted in a partial increase in cellular glycolytic capacity and acidification rate, suggesting that anaerobic metabolism within the tumor could be stimulated (Fig. 5A). However, treatment with DAF@LNPs, which contain the competitive molecule 2-DG, led to a rapid decline in glycolytic capacity. Notably, both basal oxygen consumption rate (OCR) and maximal respiration significantly increased, while ATP production slightly decreased (Fig. 5A–C). Interestingly, treatments with both AA and AF@LNPs significantly reduced cellular basal OCR, maximal respiration, and ATP production, with these effects further amplified by DAF@LNPs treatment (Fig. 5B). Collectively, these findings suggest that AA-induced mitochondrial disruption elevates tumor glycolytic energy and glucose uptake, with the inhibitory effect of the 2-DG competitive molecule becoming more apparent. At this point, with tumor mitochondria disrupted and compensatory glycolysis suppressed, the tumor confronts a state of severe energy deficiency. This necessitates the urgent production of new mitochondria, other organelles, and metabolic enzymes to transmit signals and generate energy to cope with stress sensing. At this juncture, a vast number of ribosomes are required to synthesize new proteins for replenishment.

To this end, we next investigated whether nano-energy interference could effectively disrupt the tumor's mitochondrial and ribosomal quality control systems, thoroughly impairing the tumor's stress-sensing capability, and thereby inhibiting its adaptive survival. We first evaluated the expression levels of TOM40 and S6, which serve as essential markers of mitochondria and ribosomes in tumor cells, respectively. The results indicated that only DAF@LNPs could effectively inhibit the production of mitochondria and ribosomes in the tumor (Fig. 5D). In contrast, the incomplete energy restrictions imposed by the other groups only partially inhibited the tumor's ability to generate mitochondria and ribosomes.

To confirm whether the measures of nano-energy interference to disrupt the tumor's energy metabolism and organelle quality control would impair its stress sensing capability, thereby affecting its formation and metastasis, we then employed the CCK-8 assay to evaluate the impact of various treatments on tumor cells. After a 48-h incubation period, DAF@LNPs exhibited significant cytotoxicity, resulting in only a small number of surviving cells. In contrast, the DAF@LNPs + glucose and AF@LNPs groups only showed partial inhibition of tumor cells (Fig. 5E). Further confirmation through a cell scratch assay confirmed that DAF@LNPs could effectively inhibit tumor cell migration, whereas DAF@LNPs + glucose and AF@LNPs, which solely disrupt mitochondria, could only partially inhibit tumor migration speed (Fig. 5F).

### 2.5 Tumor accumulation of nano-energy interference

**Fig. 6:**
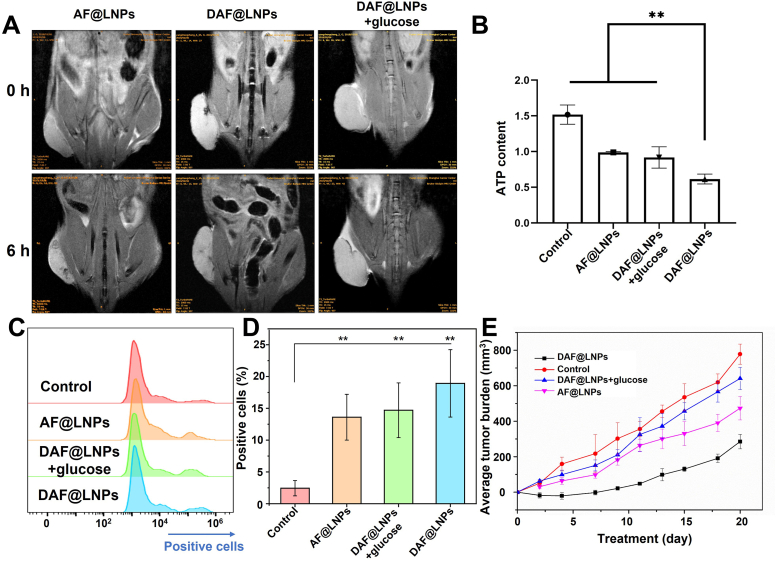
In vivo assessment of tumor-targeting ability and biodistribution of nanocomplexes. (A) T2-weighted magnetic resonance (MR) imaging of HCT116 tumor-bearing mice following intravenous injection of AF@LNPs or DAF@LNPs, respectively, at a dose of 15 mg Fe/kg body weight. (B) Quantification of ATP levels in tumors 48 h post-injection of phosphate-buffered saline (PBS), AA, AF@LNPs, or DAF@LNPs, respectively. Notably, DAF@LNPs treatment resulted in a significant reduction in ATP expression. (C) Measurement of reactive oxygen species (ROS) levels in tumors subsequent to treatment with PBS, AF@LNPs, or DAF@LNPs. DAF@LNPs treatment induced a marked increase in ROS levels. And (D) corresponding data statistical analysis. (E) Evaluation of therapeutic efficacy in tumor-bearing mice following various treatments.

As previously demonstrated, nano-energy interference possesses the profound ability to modulate tumor stress sensing, thereby hindering the growth and migration of tumor cells. Based on this premise, we posit that nano-energy interference holds the potential to enhance malignancy control within organisms and obstruct adaptive tumor growth. We subsequently assessed the in vivo tumor-targeting ability and biodistribution of this nanosystem in tumor-bearing mice. Initially, HCT116 tumor-bearing mice were intravenously injected with AF@LNPs or DAF@LNPs at a dose of 15 mg Fe/kg body weight (b.w.), followed by T2-weighted MR imaging after 6 h. Post-administration of 2-DG coupled LNPs, a marked decrease in T2 signal intensity at the tumor site was observed, more pronounced than in mice receiving non-targeted AF@LNPs (Fig. 6A). Consistent with in vitro cell experiments, co-administration with glucose led to a reduction in T2 signal intensity in the tumor area (Fig. 6A). These findings underscore the specificity and efficiency of DAF@LNPs in targeting tumors, indicating their immense potential in modulating tumor stress sensing.

### 2.6 Nano-energy interference effectively inhibits tumor growth and metastasis in vivo

Next, we further showcased the remarkable potential of nano-energy interference in effectively inhibiting tumor growth and metastasis in vivo, owing to its significant accumulation within the tumor site. Specifically, Twenty HCT116 tumor-bearing mice were divided into four groups (n = 5) and administered with either phosphate-buffered saline, AF@LNPs, DAF@LNPs, or DAF@LNPs + glucose. Intravenously injecting PBS and LNPs (15 mg Fe/kg b. w.), we collected tumors post-48-h injection for the assessment of ATP and ROS levels. As we expected, the results corroborated the in vitro observations, demonstrating a significant decrease in ATP expression (Fig. 6B) and a marked increase in ROS levels following DAF@LNPs treatment (Fig. 6C and D). This evidence underscores the ability of NEI to induce tumor homeostasis imbalance in the in vivo environment.

**Fig. 7:**
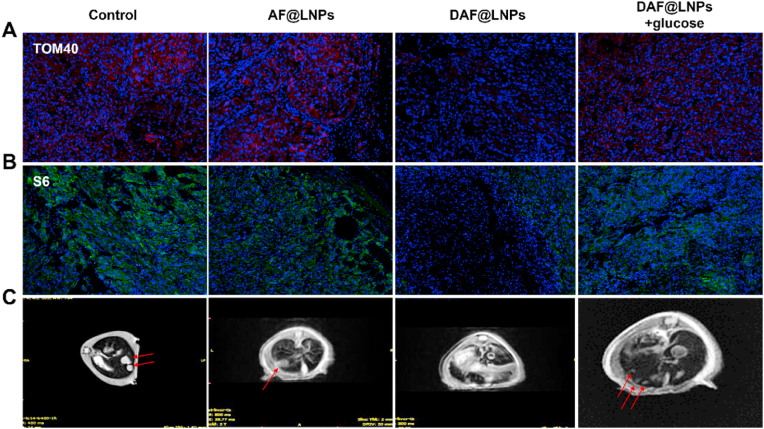
Pathological staining and metastasis inhibition by DAF@LNPs. (A) Immunohistochemical staining of tumor tissues seven days post-treatment, demonstrating the expression levels of TOM40 and S6. (B) Immunohistochemical staining of tumor tissues depicting the expression levels of S6, a pivotal marker of ribosomes. (C) Establishment of a colorectal cancer liver metastasis model in mice, achieved by intrasplenic injection of homologous HCT116 colorectal cancer cells.

Tumor adaptive survival relies on factors such as nutrient acquisition, adequate energy metabolism, and protein neogenesis. Despite the development of numerous targeted treatments based on these factors, passive strategies stemming from the complex evolutionary pathways of tumors continue to face challenges in effectively eradicating tumors. Consequently, we propose that proactive management of tumor stress-sensing processes may yield unexpected results (Fig. 6E). To validate our hypothesis, we conducted pathological staining of tumor tissues seven days post-treatment. We assessed the expression levels of TOM40 and S6, key markers of mitochondria and ribosomes in tumor tissues, respectively. The results revealed that only DAF@LNPs could effectively inhibit the production of mitochondria and ribosomes in tumors (Fig. 7A and B). In contrast, the remaining groups either imposed incomplete energy restrictions or failed to prevent the generation of tumor ribosomes, partially restoring their energy and protein synthesis capabilities.

Metastasis significantly impacts tumor recurrence and mortality rates, posing a substantial challenge in the field of tumor treatment. To address this, we established a colorectal cancer liver metastasis model in mice by injecting homologous HCT116 CRC cells into the spleen (Fig. 7C). The HCT116 cells could then migrate to the liver through the bloodstream, making this model crucial for studying the mechanisms of malignant tumor liver metastasis and anti-metastasis treatments. Leveraging the exceptional MRI imaging capabilities of DAF@LNPs, we observed that DAF@LNPs effectively prevented the formation of liver metastasis foci compared to the control group (Fig. 7C and Supplementary Fig. S4). AF@LNPs and DAF@LNPs + glucose were able to inhibit the quantity and size of metastatic tumors partially. These findings unequivocally establish the inhibitory effect of DAF@LNPs on adaptive tumor changes and metastasis formation, highlighting the innovative potential of NEI in cancer treatment.

Mitochondria play a crucial role in the energy production, metabolic remodeling, and signal transduction of tumor cells, essential for supporting tumor growth and metastasis. Recent research advances indicate that during the process of metastasis, tumor cells undergo metabolic remodeling to adapt to the continuously changing microenvironment [[18], [19], [20]]. This involves enhanced mitochondrial biogenesis and respiration, as well as adjustments in ribosome production to meet the demands of protein synthesis. These metabolic adaptation strategies enable tumor cells to survive under conditions of limited energy supply and establish metastatic foci in new tissue environments.

DAF@LNPs inhibit tumor metastasis by targeting these critical metabolic pathways of tumor cells. Firstly, by disrupting the mitochondrial respiratory chain with succinic acid, DAF@LNPs weaken the energy production capacity of tumor cells. Simultaneously, the addition of 2-DG further restricts the dependence of tumor cells on glucose, a crucial fuel source in their metabolic remodeling process. This dual attack not only limits the energy production of tumor cells but also hinders their necessary protein synthesis under stress conditions, as ribosome generation is inhibited.

In summary, DAF@LNPs hinder tumor cells' adaptation and survival in new environments by disrupting mitochondrial function and inhibiting the energy metabolism of tumor cells, thereby suppressing tumor growth and metastasis. The highlight of this strategy lies in controlling the invasiveness and metastatic capability of tumors by targeting the metabolic characteristics and stress response mechanisms of tumor cells.

## 3 Summary and perspective

**Fig. 8:**
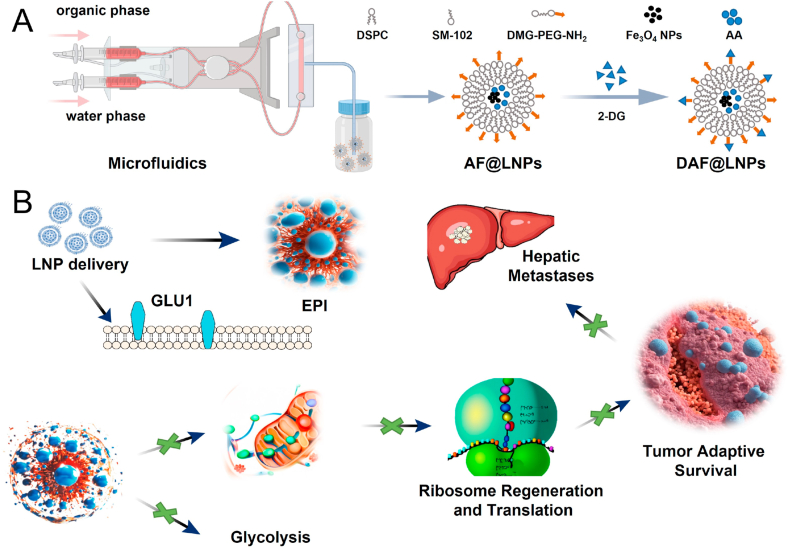
Schematic model of a lipid nanoparticle-based nano-energy interference (NEI) device designed to disrupt tumor energy metabolism. (A) A schematic diagram of LNPs synthesis using microfluidics. And (B) the assumed working principle inside cells. The device encapsulates azelaic acid, augmenting mitochondrial disruption and intensifying tumor energy deficiency. Additionally, the device binds with the glucose analog, 2-deoxy-D-glucose, inhibiting tumor glucose uptake. The sustained release of azelaic acid induces comprehensive energy deficiency in tumors, impeding ribosome generation. This innovative strategy presents a promising approach to the treatment of tumors with spontaneous metastasis.

In this study, we introduced a novel approach to cancer treatment, termed nano-energy interference, which effectively disrupted tumor adaptation and metastasis by inducing an energy crisis within the tumor microenvironment (Fig. 8). The innovative NEI strategy relied on manipulating tumor stress-sensing mechanisms, representing a proactive departure from traditional passive treatments. However, the NEI strategy employed a cyclically amplified nano-energy interference device capable of not only efficiently targeting tumors but also monitoring their status. The achievement of the therapeutic effect on tumors was realized through the simultaneous inhibition of mitochondrial respiration and biogenesis, as well as ribosome production, thereby eventually effectively controlling the proliferation and hepatic metastasis of colon cancer in vivo.

These findings demonstrated that the NEI device could induce a comprehensive energy deficiency in tumors by disrupting the mitochondrial respiratory chain and blocking the generation of energy-sensitive ribosomes. Consequently, tumors lose their energy supply and new protein generation, rendering them incapable of effectively coping with environmental stress, thereby inhibiting the growth and metastasis of tumors.

This study underscored the potential of NEI as a promising therapeutic strategy for refractory tumors. However, it is worth noting that while our results are promising, further research is necessary to fully understand the long-term effects and potential side effects of NEI treatment. Moreover, further exploration into the optimal dosage and delivery methods of the NEI device is warranted to maximize its therapeutic efficacy while minimizing potential adverse effects. In conclusion, our study presents a paradigm shift in the approach to cancer treatment, with the potential to impact the field of oncology significantly. The innovative NEI strategy represents a promising therapeutic avenue for refractory tumors, providing a novel approach to combat the adaptive survival and metastasis of tumors. Future research should focus on optimizing the NEI device and validating its efficacy across a broader range of tumor types, with the ultimate goal of translating this novel therapeutic strategy into clinical application.

## Supporting Information

Supporting Information is available from the online or from the author.

## CRediT authorship contribution statement

**Fei Teng:** Writing – original draft, Validation, Software, Resources, Methodology, Formal analysis, Data curation. **Dong Fu:** Writing – review & editing, Writing – original draft, Visualization, Software, Methodology, Investigation, Formal analysis, Conceptualization. **Chen-Cheng Shi:** Writing – original draft, Resources, Methodology, Formal analysis, Data curation. **An Xiong:** Writing – original draft, Validation, Software, Formal analysis. **Meng-Xuan Yang:** Software, Methodology, Formal analysis. **Chang Su:** Visualization, Validation, Software. **Ming Lei:** Validation, Software, Methodology. **Yi-Ou Cao:** Writing – original draft, Software, Investigation. **Xiao-Dong Shen:** Validation, Resources, Methodology. **Yi Chen:** Writing – review & editing, Project administration, Conceptualization. **Pu-Hua Wang:** Validation, Supervision, Resources, Conceptualization. **Shao-Qun Liu:** Writing – review & editing, Supervision, Resources, Project administration, Funding acquisition, Conceptualization.

## Declaration of competing interest

The authors declare that they have no known competing financial interests or personal relationships that could have appeared to influence the work reported in this paper.

## Data Availability

Data will be made available on request.
