# Supplementary material for: Nano-energy interference: A novel strategy for blunting tumor adaptation and metastasis
Source: Mater Today Bio. 2024 Feb 3;25:100984. doi: 10.1016/j.mtbio.2024.100984 (PMC10865032; doi:10.1016/j.mtbio.2024.100984)
Supplement: Multimedia component 1 [file mmc1.docx]

**Supporting Information**

**Nano-Energy Interference: A Novel Strategy for Blunting Tumor Adaptation and Metastasis**

Fei Teng^1,2†^, Dong Fu^3†^, Chen-Cheng Shi^1,2†^, An Xiong^1,2^, Meng-Xuan Yang^1,2^, Chang Su^1,2^, Ming Lei^1,2^, Yi-Ou Cao^1,2^, Xiao-Dong Shen^1,2^, Yi Chen^1,2*^, Pu-Hua Wang^1,2*^, Shao-Qun Liu^1,2*^

^1^ Department of Gastrointestinal Surgery, Minhang Hospital, Fudan University, Shanghai, 201199, P. R. China.

^2^ Key Laboratory of Whole-period Monitoring and Precise Intervention of Digestive Cancer (SMHC), Minhang Hospital & AHS, Fudan University, Shanghai, China.

^3^ Department of Pediatric Orthopedics, Children's Hospital of Fudan University, National Children’s Medical Center, Shanghai 201102, China.

^†^ These authors contributed equally to this work.

^*^ Correspondence: liushaoqun@fudan.edu.cn; kydding@sjtu.edu.cn; chen_yi@fudan.edu.cn

**Materials and Methods**

**Synthesis of DAF@LNPs**

The nano-energy interference (DAF@LNPs) was synthesized employing a microfluidic technology as previously reported^[1]^. Briefly, a lipid mixture comprising phospholipids, cholesterol, and PEG-lipid was first prepared. Fe_3_O_4_ nanoparticles and azelaic acid (AA) were then co-loaded into the lipid nanoparticles. Subsequently, modification with 2-deoxy-D-glucose (2-DG) was accomplished by incorporating 2-DG-modified PEG-lipid into the lipid mixture. The synthesis was conducted using a Nano Assemblr Benchtop system (Precision NanoSystems) to facilitate controlled mixing and nanoparticle formation.

**Characterization of DAF@LNPs**

Transmission electron microscopy (TEM) analysis was conducted using a JEOL JEM-2100F transmission electron microscope operating at 200 kV. Samples were prepared by drop-casting a diluted DAF@LNPs suspension onto a carbon-coated copper grid, followed by negative staining with uranyl acetate. The morphology and size distribution of DAF@LNPs were subsequently observed and analyzed. Dynamic light scattering analysis was performed using a Malvern Zetasizer Nano ZS equipped with a 633 nm laser. DAF@LNPs suspensions were appropriately diluted in phosphate-buffered saline and subjected to examination at 25°C to determine the size distribution and polydispersity index.

**Loading Efficiency Determination**

The loading efficiencies of iron and AA in DAF@LNPs were assessed utilizing a PerkinElmer Analyst 800 atomic absorption spectrometer for iron and a Shimadzu HPLC system equipped with a UV-Vis detector for AA. Calibration curves were established using standard solutions of iron and AA, and loading efficiencies were calculated based on the concentrations of iron and AA in the DAF@LNPs formulation.

**Stability Analysis**

The stability of DAF@LNPs was examined through dynamic light scattering analysis. DAF@LNPs suspensions were prepared in the cell culture medium containing 10% FBS and stored at 37°C. DLS measurements were conducted at predetermined intervals over 14 days to track changes in hydrodynamic size and polydispersity index.

**Magnetic Resonance Imaging (MRI) Evaluation**

The MRI capabilities of DAF@LNPs were assessed using T2-weighted MR imaging on a Siemens MAGNETOM Skyra 3T MRI scanner. Firstly, DAF@LNPs suspensions at various concentrations were positioned in sample tubes and imaged using a T2-weighted sequence. Then the transverse relaxation rate (r2) was calculated based on the signal intensity of the MR images and the concentration of DAF@LNPs.

**Preparation of Comparison Samples**

Lipid nanoparticles lacking 2-deoxy-D-glucose (2-DG) modification (AF@LNPs) and fluorescently labeled lipid nanoparticles (Fluro-DAF@LNPs) were synthesized using comparable methods for subsequent biological investigations. The characterization and evaluation of these reference samples were performed in parallel with DAF@LNPs.

**Cell Culture**

HCT116, a colon cancer cell line characterized by elevated glucose transporter-1 (GLUT-1) expression and glucose consumption, was cultured in McCoy's 5A medium (Thermo Fisher Scientific, Cat. No. 16600082) supplemented with 10% fetal bovine serum (Thermo Fisher Scientific, Cat. No. 10437028) and 1% penicillin-streptomycin (Thermo Fisher Scientific, Cat. No. 15140122). Cells were maintained at 37°C in a humidified atmosphere with 5% CO2.

**Synthesis of FITC-Labeled Lipid nanoparticles**

FITC-labeled lipid nanoparticles (Fluro-DAF@LNPs and Fluro-AF@LNPs) were synthesized according to a previously established protocol^[2]^. In brief, lipids (Avanti Polar Lipids, Cat. No. 840457) and FITC (Thermo Fisher Scientific, Cat. No. F1906) were co-dissolved in chloroform and evaporated to form a thin film. The film was hydrated with a buffer solution and then extruded through a polycarbonate membrane to obtain FITC-labeled LNPs.

**Confocal Imaging**

HCT116 cells were cultured in confocal dishes and treated with FITC-labeled LNPs (Fluro-DAF@LNPs and Fluro-AF@LNPs) at a concentration of 50 µg Fe/mL for 3 hours. Confocal imaging was conducted using a Zeiss LSM 800 confocal laser scanning microscope equipped with appropriate filters for FITC and DAPI. Co-localization studies were carried out by co-staining cells with MitoTracker Red (Thermo Fisher Scientific, Cat. No. M7512) to determine the spatial relationship between LNPs and mitochondria.

**Flow Cytometry**

Flow cytometry analysis was executed to validate the specific uptake and internalization of Fluro-DAF@LNPs in HCT116 cells. Cells were exposed to FITC-labeled LNPs, and flow cytometry data were acquired using a BD LSRFortessa flow cytometer.

**Transcriptomics Analysis**

Total RNA extraction from tumor cells was accomplished using the RNeasy Mini Kit (Qiagen, Cat. No. 74106) in accordance with the manufacturer's protocol. The quality and quantity of RNA were evaluated using a NanoDrop spectrophotometer, respectively. Transcriptomic analysis was conducted utilizing the Illumina HiSeq platform, and differential gene expression was assessed using the DESeq2 package in R software.

**Venn Diagram and Volcano Plot**

The generation of the Venn diagram and volcano plot was performed using R software (version 4.0.3) and the ggplot2 package for effective visualization.

**Gene Ontology (GO) Enrichment Analysis**

GO enrichment analysis was executed utilizing the cluster Profiler package in R software to investigate the impact of DAF@LNPs treatment on biological processes, cellular components, and molecular functions respectively.

**Mitochondrial Status Assessment**

Tumor cells respectively treated with AA, AF@LNPs, or DAF@LNPs were assessed for mitochondrial status using JC-1 staining. Fluorescence microscopy images were captured with a Zeiss Axio Observer microscope (Model: Axio Observer) equipped with appropriate filters.

**Cellular Electron Microscopy**

Tumor cells were fixed and processed for electron microscopy imaging using a JEOL JEM-1400 transmission electron microscope operating at 120 kV.

**Reactive Oxygen Species (ROS) Assessment**

Intracellular ROS levels were determined using 2′,7′-dichlorodihydrofluorescein diacetate (DCFH-DA) staining. Fluorescence microscopy images and flow cytometry analysis were performed with a BD FACSCanto II flow cytometer.

**Intracellular Glucose Measurement**

Intracellular glucose levels were quantified using a glucose assay kit (Abcam Glucose Assay Kit, Cat. No. ab65333) following treatment with AA, AF@LNPs, or DAF@LNPs, respectively. Absorbance measurements were conducted using a microplate reader (BioTek Synergy H1 Hybrid Multi-Mode Reader) at the appropriate wavelength.

**Seahorse XFe96 Analyzer Profiling**

Tumor cells were treated with AA, AF@LNPs, or DAF@LNPs, and bioenergetic profiling was executed using the Seahorse XFe96 Analyzer. Measurements of glycolytic capacity, acidification rate, basal oxygen consumption rate (OCR), maximal respiration, and ATP production were evaluated according to the manufacturer's guidelines.

**Immunoblot Analysis**

Evaluation of TOM40 and S6 expression levels in tumor cells post-treatment with AA, AF@LNPs, or DAF@LNPs was performed, respectively. Protein samples were separated by SDS-PAGE and transferred to a PVDF membrane for immunoblotting utilizing specific antibodies (e.g., TOM40 antibody, S6 antibody, and appropriate loading controls). Imaging and quantification were carried out using a chemiluminescence imaging system (Bio-Rad ChemiDoc Imaging System).

**Cell Viability Assessment**

Cell viability was determined via the CCK-8 assay following a 48-hour incubation period with AA, AF@LNPs, or DAF@LNPs, respectively. Absorbance was measured at the appropriate wavelength using a microplate reader (BioTek Synergy H1 Hybrid Multi-Mode Reader).

**Cell Scratch Assay**

Evaluation of tumor cell migration was conducted using a cell scratch assay post-treatment with AA, AF@LNPs, or DAF@LNPs, respectively. Images were captured at specific time points using a phase-contrast microscope (Zeiss Axio Observer microscope) equipped with appropriate imaging software for subsequent analysis.

**In Vivo Tumor-Targeting and Biodistribution of Nanosystem Assessment**

HCT116 tumor-bearing mice were intravenously administered AF@LNPs or DAF@LNPs at a dose of 15 mg Fe/kg body weight. After 6 hours, T2-weighted MR imaging was performed using a 7T MRI scanner (Bruker BioSpec 70/20 USR) to evaluate the tumor-targeting ability of the nano complexes.

**Pathological Staining and Marker Expression Analysis**

Tumor tissues were collected post-seven days of treatment with DAF@LNPs, AA, and AF@LNPs, respectively, and PBS-treated mice as the controls. Immunohistochemical staining was conducted to assess the expression levels of TOM40 and S6 using specific antibodies (TOM40 antibody: Abcam, Cat. No. ab186734; S6 antibody: Cell Signaling Technology, Cat. No. 2317S). Stained tissue sections were imaged using a digital pathology scanner (Leica Aperio AT2) and analyzed for marker expression levels.

**Establishment of Colorectal Cancer Liver Metastases Models**

The colorectal cancer liver metastases models were established by intrasplenic injection of homologous HCT116 colorectal cancer cells into mice. The formation of liver metastasis foci was assessed through MRI imaging using a 7T MRI scanner (Bruker BioSpec 70/20 USR) following treatment with DAF@LNPs, AA, AF@LNPs, or PBS, respectively. All animal procedures in this study were conducted in adherence to the guidelines approved by the Institutional Animal Care and Use Committee (IACUC) of Fudan University (2023-MHFY-18J2S).

**Statistical Analysis**

Quantitative data were presented as mean ± standard deviation from a minimum of three independent experiments. Statistical significance was determined using Student's t-test, with p < 0.05 considered as statistically significant.

**References**

1. A. Le Bras, Nanoparticles for pulmonary RNA delivery, Lab Anim. 52(5) (2023) 100, https://doi.org/10.1038/s41684-023-01170-9.
2. S.L. Dai, S.J. Wu, N. Duan, Z.P. Wang, A near-infrared magnetic aptasensor for Ochratoxin A based on near-infrared upconversion nanoparticles and magnetic nanoparticles, Talanta 158 (2016) 246-253, https://doi.org/10.1016/j.talanta.2016.05.063.


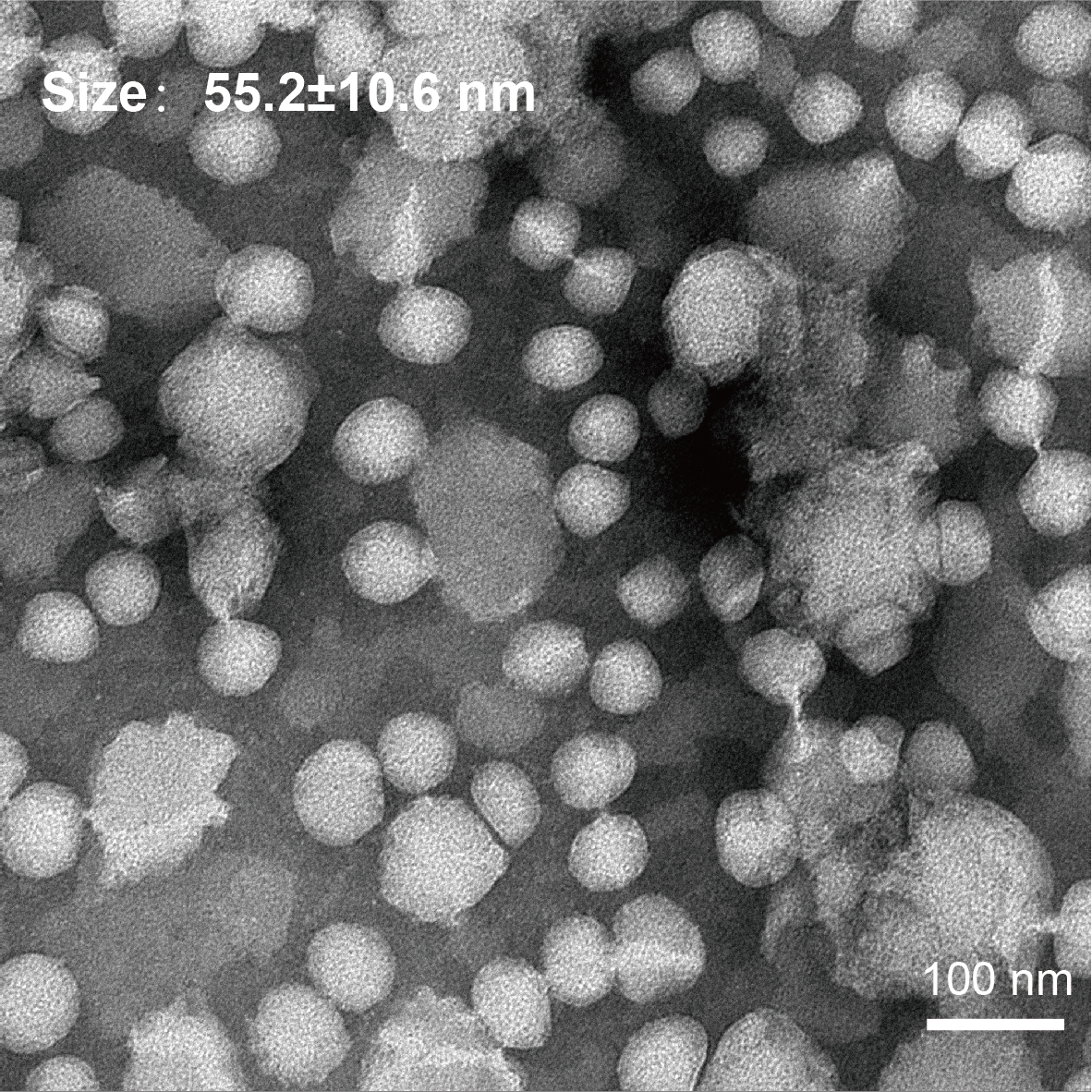


**Fig. S1.** Transmission electron microscopy (TEM) of DAF@LNPs. TEM image of DAF@LNPs was acquired subsequent to negative staining with tungstophosphoric acid. Subsequently, ImageJ software was employed for statistical analysis of particle size distribution, revealing an average diameter of 55.2±10.6 nm.


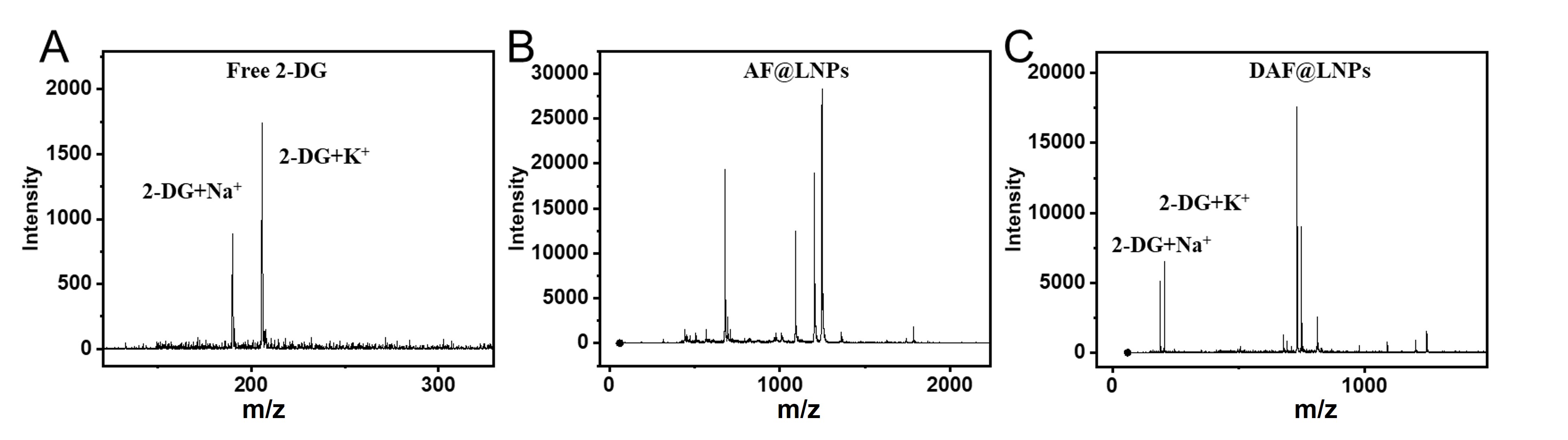


**Fig. S2.** Characterization of 2-DG on DAF@LNPs. The successful modification of 2-DG onto LNPs was confirmed via mass spectrometry. Panel (A, C) illustrated the presence of characteristic peaks for the free small molecule 2-DG in DAF@LNPs, offering a distinct contrast as these peaks were absent in AF@LNPs (B).


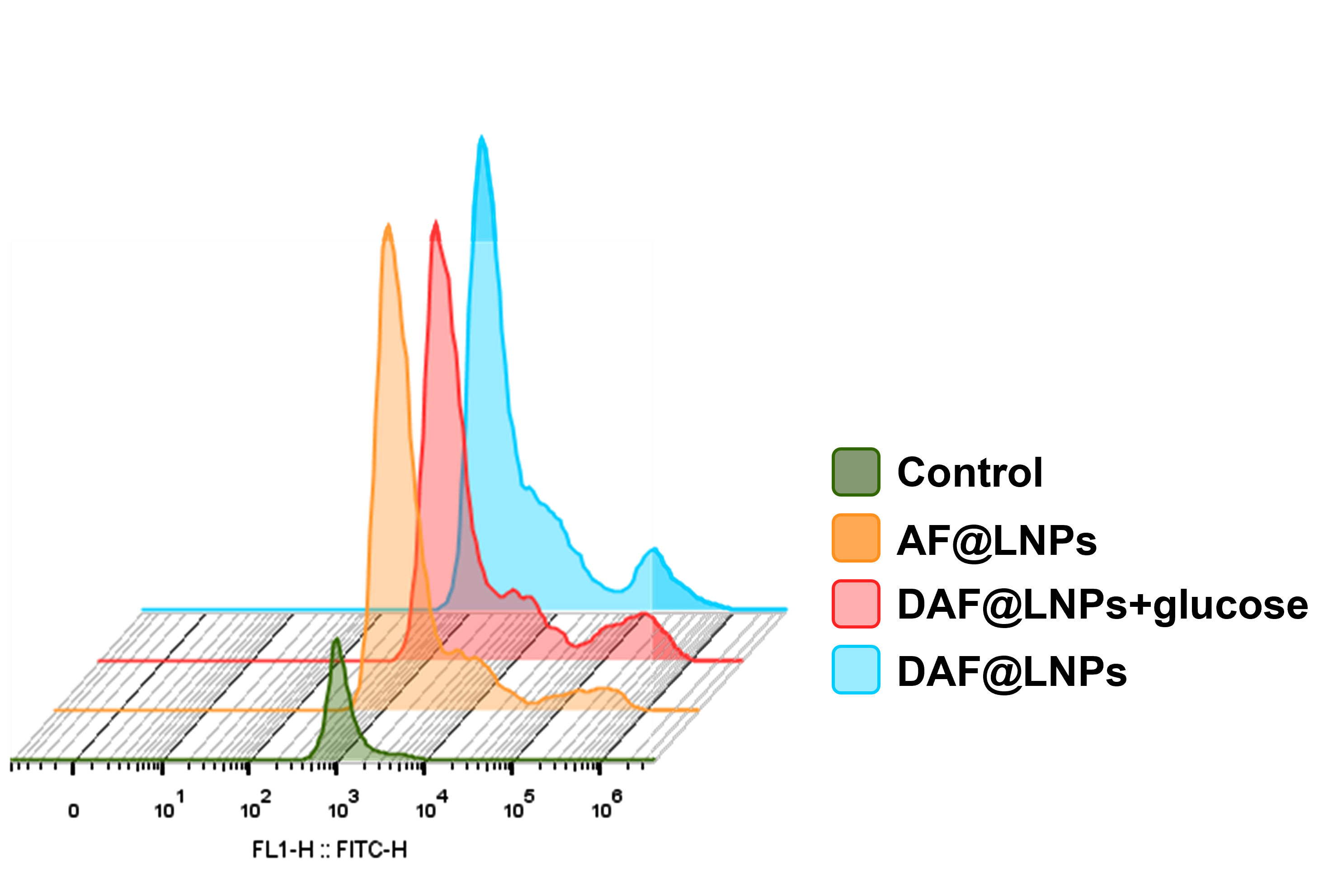


**Fig. S3.** Validation of the internalization of Fluro-DAF@LNPs in HCT116 cells was conducted through flow cytometry. The confirmation of targeted cellular uptake and internalization of Fluro-DAF@LNPs in HCT116 cells was achieved using flow cytometry. The experimental protocol comprised a 3-hour incubation with FITC-labeled LNPs (50 µg Fe/mL), including distinct conditions for Fluro-DAF@LNPs, the non-targeted Fluro-AF@LNPs group, and the introduction of additional glucose, respectively.


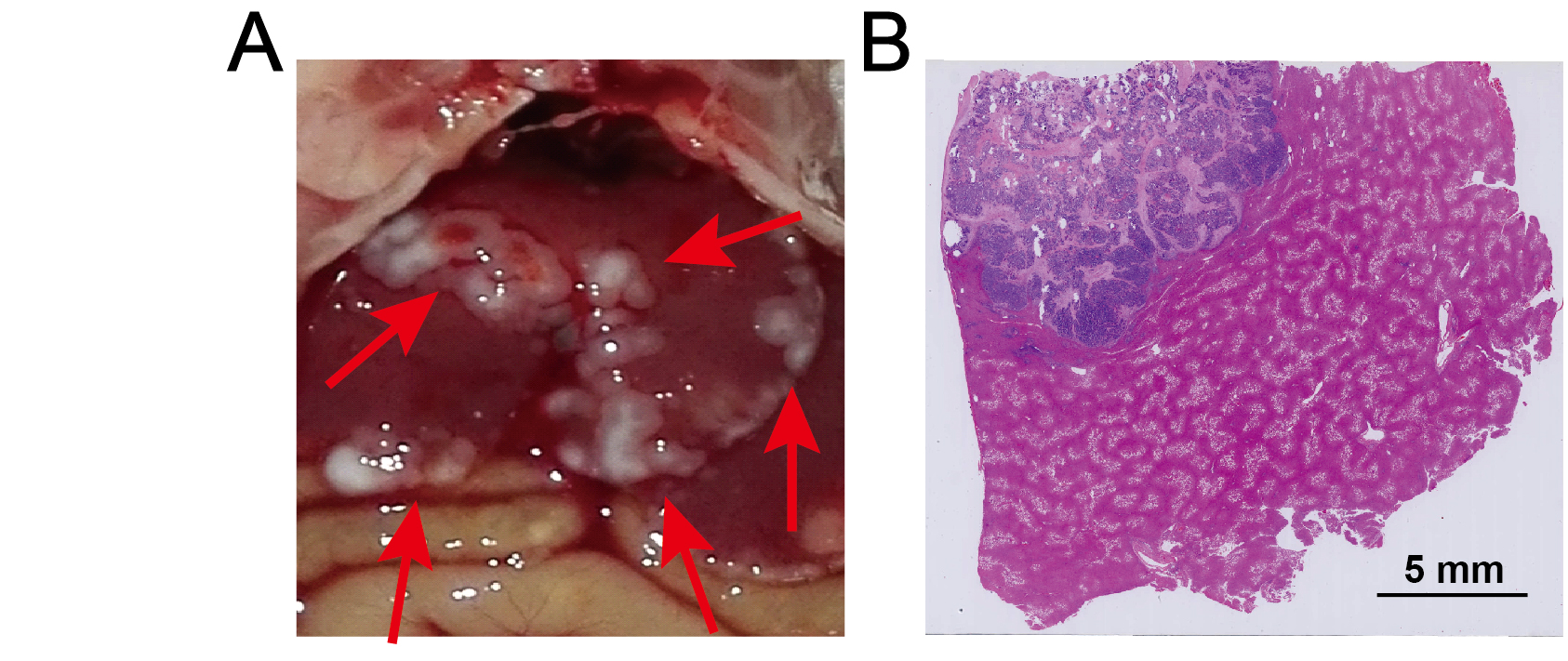


**Fig. S4.** Examination of mouse livers to assess the presence of metastatic lesions at the treatment endpoint in the control group. (A) Bright-field images clearly revealed multiple tumor nodules on the liver surface. (B) Additionally, H&E-stained liver tissue slices prominently exhibited this phenomenon. Red arrows in the pictures indicate the locations of metastatic nodules.

**Table S1.** Gene enrichment abbreviations.

| **Abbreviation** | **Full name** |
| --- | --- |
| EMO  ESO  AFO  MRCCA  ATP MP  PRMP  PRTMP  C-SA  GPME  AG | extracellular matrix organization  extracellular structure organization  actin filament organization  mitochondrial respiratory chain complex assembly  ATP metabolic process  purine ribonucleotide metabolic process  purine ribonucleoside triphosphate metabolic process  cell-substrate adhesion  generation of precursor metabolites and energy  angiogenesis |
| EM  PEM  MPC  EMC  AC  MS  MIM  OR  MR  MM | extracellular matrix  proteinaceous extracellular matrix  mitochondrial protein complex  extracellular matrix component  actin cytoskeleton  myelin sheath  mitochond drial inner membrane  organellar ribosome  mitochondrial ribosome  mitochondrial matrix |
| SMA  CAMB  AB  AFB  EMSC  CB  SCC  IB  CB  HSPB | structural molecule activity  cell adhesion molecule binding  actin binding  actin filament binding  extracellular matrix structural constituent  cadherin binding  structural constituent of cytoskeleton  integrin binding  collagen binding  heat shock protein binding |
| GMP  HMP  HM  CCM  RGMP  GMP  MCP  RCMP  CCMP  MMP | glucose metabolic process  hexose metabolic process  histone modification  covalent chromatin modification  regulation of glucose metabolic process  glycerolipid metabolic process  muscle cell proliferation  regulation of carbohydrate metabolic process  cellular carbohydrate metabolic process  monosaccharide metabolic process |
